# Effects of Vitamin C Supplementation on Tetrabenazine Treatment in a Caenorhabditis elegans Huntington’s Disease Model

**DOI:** 10.17912/micropub.biology.002193

**Published:** 2026-06-27

**Authors:** Brenda Tinoco-Bravo, Jacob Manjarrez

**Affiliations:** 1 Biochemistry and Microbiology, Oklahoma State University Center for Health Sciences, Tulsa, OK, United States

## Abstract

Tetrabenazine, a VMAT2 inhibitor, is widely used to treat chorea in Huntington's disease by depleting monoamine neurotransmitters, particularly dopamine. However, this mechanism may indirectly increase oxidative stress due to enhanced metabolism and auto‑oxidation of cytosolic monoamines, generating reactive oxygen species. Using Caenorhabditis elegans polyglutamine models, this study investigated the effects of tetrabenazine and vitamin C supplementation on survival. These results indicate that tetrabenazine reduces survival, while vitamin C, a known antioxidant, mitigates these effects and improves outcomes, particularly in pathogenic poly‑Q strains. Our findings suggest that antioxidant supplementation may counteract tetrabenazine‑associated oxidative stress and enhance therapeutic efficacy in neurodegenerative disease.

**Figure 1. Tetrabenazine treatment with Vitamin C supplementation in a C. elegans HD model:** Only the highest significance level according to the Mantel-Cox and/or Gehan-Breslow-Wilcoxon tests is indicated in the figures. The median tables state the lifespan at 50% survival, and the dotted line is a visual indicator.&nbsp; The p-value distribution follows the p > 0.05 (*), p > 0.01 (**), p > 0.001 (***), p > 0.0001 (****) (GraphPad, Dotmatics, version 11). Dimethyl sulfoxide (DMSO); Tetrabenazine (100 µM) (TBZ); Vitamin C (1mM) (Vit C); Tetrabenazine (100 µM) + Vitamin C (1mM) (TBZ + Vit C). The n=animals A) AM134 : Vit C (0.5 mM) 58, Vit C (1mM) 52, NGM 61. B) EAK102 : 57, 68, 60. C) EAK103 : 48, 67, 39 D) AM134 : DMSO 165, TBZ 172, Vit C 197, TBZ + Vit C 196. E) EAK102 : 161, 169, 181, 205. F) EAK103 : 202, 174, 191, 175.

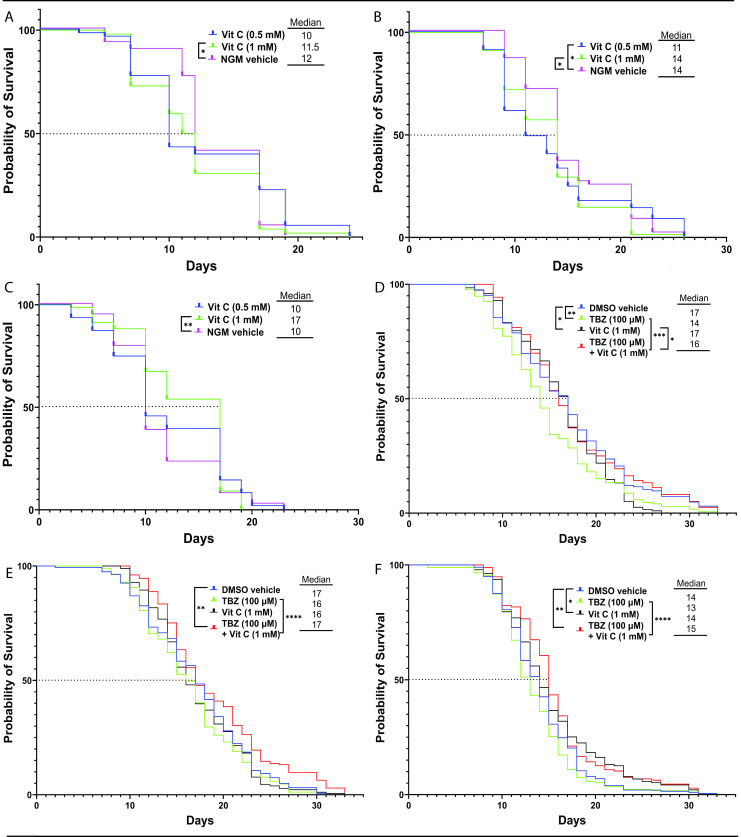



## Description

Involuntary movements, or chorea, are common features in neurodegenerative disorders as these diseases progress. In the treatment of Huntington's disease (HD), tetrabenazine is a useful and regularly prescribed pharmaceutical (Dalton et al., 2024). Tetrabenazine functions as a vesicular monoamine transporter‑2 (VMAT2) inhibitor (Dalton et al., 2024).&nbsp; Its primary role is to reduce levels of monoamine neurotransmitters in the brain, particularly dopamine in the case of Huntington's disease (Wu et al., 2024). By inhibiting VMAT2, monoamines are prevented from entering synaptic vesicles, leading to their accumulation in the cytoplasm, where they are subsequently broken down by monoamine oxidases (MAO) (Wu et al., 2024). By lowering dopamine levels, tetrabenazine has been shown to help control abnormal hyperkinetic movements (Szpisjak et al., 2020).&nbsp;

However, tetrabenazine may be indirectly associated with the production of reactive oxygen species (ROS), rather than through its primary pharmacological function. Monoamine neurotransmitters that remain in the cytosol are metabolized by MAO, generating hydrogen peroxide, a relatively long-lived ROS, as a by-product. This process occurs both within and outside the mitochondria, where MAO is localized (Wang et al., 2013). In addition, free cytosolic dopamine can undergo auto-oxidation, leading to the formation of quinones and other ROS (Meiser et al., 2013; Kawano et al., 2002). Collectively, these mechanisms suggest that tetrabenazine may indirectly contribute to increased oxidative stress by inhibiting VMAT2, resulting in cytosolic accumulation of monoamines such as dopamine, which are subsequently metabolized and generate ROS, including H₂O₂ (Crawford RM, Anderson EJ, 2025).

*Caenorhabditis elegans* possesses three genes that appear to represent this group: *amx-1* , *amx-2* , and *amx-3* . However, based on sequence homology, *amx-2* is more closely related to human monoamine oxidases MAO-A and MAO-B (Wang et al., 2017). These genes encode enzymes that oxidize monoamines and function similarly to their mammalian counterparts (Wang et al., 2017). Loss of function in *amx-2* alters monoamine levels in the worm, which in turn affects locomotion, feeding, and stress-response behaviors (Wang et al., 2017; Donnelly et al., 2013; Ferkey et al., 2021). However, there is currently no direct association for vitamin C in the reduction of ROS produced by MAO (Ward et al., 2013). Vitamin C acts as a strong antioxidant, which can modulate the reactive byproducts produced through the activity of MAO (Ward et al., 2013; Gęgotek, A., & Skrzydlewska, E, 2022; Crawford RM, Anderson EJ, 2025). This highlights MAO as a valuable target for studying the neurobiology of monoamine-related behaviors in a simple, genetically tractable organism.

Using the Gehan-Breslow-Wilcoxon test and focusing on early events in a preliminary analysis, an appropriate vitamin C (Vit C) concentration was determined. A control for the fluorescent protein ( Figure 1A ), AM134 (YFP:: **Q0** ), grown on NGM, shows a significant difference in survival compared to Vit C at 1 mM. However, overall lifespan favors the 1 mM condition; this difference is not statistically significant. In the non-pathogenetic poly-Q repeat range Huntingtin (Httn) EAK102 (YFP::Httn513( **Q15** )), both Vit C concentrations show significant variation in early-life events, favoring Vit C ( Figure 1B ). The extended poly-Q tract strain EAK103 (YFP::Httn513( **Q128** )) shows a significant effect favoring Vit C over the NGM control; however, this does not extend to overall survival, as shown by the Mantel-Cox test ( Figure 1C ).

The full Q0 survival analysis measured the impact of TBZ supplemented with vitamin C at 1 mM, to determine if there are significant effects without the Httn protein. The Q0 TBZ condition shows a significant decrease in survival in both short-term and distributed survival compared to its DMSO vehicle control ( Figure 1D ). The Q0 Vit C condition shows a significant decrease in distributed survival, but no change in short-term survival. The combined TBZ/Vit C treatment shows a significant increase in both tests compared to TBZ alone, and a significant difference in distributed survival compared to the Vit C condition ( Figure 1D ).

For Q15, which represents the non-pathogenetic poly-Q range in HD (Lee et al., 2012), the combination treatment has significant effects on both survival distribution and short-term survival compared to DMSO and TBZ ( Figure 1E ). In the Q128 model, representing the extended repeat range implicated in Huntington's disease (Lee et al., 2012), both Vit C and the combination treatments significantly improved survival distribution compared to DMSO. However, only the combination treatment showed a significant effect on short-term survival ( Figure 1F ).

Overall, the controls Q0 (YFP) and Q15 (non-pathogenetic repeat range Httn) are comparable in overall survival, but with TBZ showing a more pronounced median lifespan effect.&nbsp; The combined treatment (Vit C + TBZ) shows a significant difference compared to TBZ alone in both the normal-range Httn (Q15) and the expanded-repeat Httn (Q128), suggesting that vitamin C supplementation may ease some of the adverse effects associated with TBZ treatment in the HD models.

The results indicate that TBZ, while a widely used and effective treatment for chorea in neurodegenerative diseases such as HD and other movement disorders, may negatively impact survival in this treatment population. In contrast, Vit C, a well-established antioxidant, appears to alleviate this effect and may even enhance survival when co-administered with TBZ, particularly in populations with abnormal poly-Q expansions. Antioxidants have been shown to mitigate oxidative stress associated with impaired proteostasis (Chen et al., 2024). Notably, HD is characterized by elevated oxidative stress (Zheng et al., 2018) and is strongly associated with mitochondrial dysfunction, which is a hallmark of many neurodegenerative diseases (Liu et al., 2025). These interconnected processes contribute to increased protein aggregation and further exacerbate oxidative damage.

TBZ may indirectly contribute to oxidative stress through inhibition of VMAT2, which leads to increased cytosolic monoamines such as dopamine. These monoamines undergo enzymatic degradation and auto-oxidation, generating reactive oxygen species (ROS). The extent of ROS production resulting from TBZ treatment warrants further investigation in future studies. However, Vit C may counteract these effects by acting as an antioxidant that donates electrons to neutralize ROS and thereby preventing oxidative damage (Conklin et al., 2024). This protective mechanism may support improved survival outcomes, particularly in pathogenic models such as the Q128 strain treated with TBZ.

## Methods

Culturing *Caenorhabditis elegans*

*C. elegans* were cultured at 20°C on nematode growth medium (NGM) agar. Plates were seeded with E. coli strain OP50 and grown overnight at 20°C.&nbsp; *C. elegans* were age-synchronized using the egg lay technique on NGM plates and incubated at 20°C until worms reached the L4 stage. At L4, all worms were moved to vitamin or combinatory condition plates and monitored throughout the lifespan.

**Table:** 

Strain Name	Genotype	Referred to as
AM134	rmIs134 [unc-54p::Q0::YFP]	Q0
EAK102	eeeIs1 [unc-54p::Htt513(Q15)::YFP::unc-45 3'UTR]	Q15
EAK103	eeeIs2 [unc-54p::Htt513(Q128)::YFP::unc-45 3'UTR]	Q128

Vitamin and tetrabenazine supplementation

The concentration of vitamin C, tetrabenazine, and the combination used were made according to the following recipe.&nbsp; The incorporation of vitamin C (Fisher Bioreagents) and/or tetrabenazine (Thermo Scientific) was made in 500 mL for 60 mm plates. All vitamin concentrations were prepared in 1 mM stock solutions using 500 L of autoclaved, deionized water, and 100 µM tetrabenazine was prepared in DMSO (Fisher Bioreagents) for combination plates. For the vitamin C-only plate condition, DMSO was added to the media containing the dissolved vitamins to incorporate DMSO across all conditions.

Survival analysis

All strains were age-synchronized using the egg lay technique and incubated at 20°C until all worms reached the L4 stage. All condition plates were maintained at 20°C throughout the survival assay, and worms were transferred to fresh plates every 2 days. Worm deaths were recorded by prodding for worm movement using a platinum worm pick, and any other events (avid, matricide, etc.) were censored from counts and analysis. Three biological replicates were performed for each strain and condition plate, with ~25 worms/plate for analysis.
